# Toward a mixed-methods framework for evaluating digital health systems in low-resource settings: an infrastructure-aware perspective

**DOI:** 10.3389/fdgth.2026.1860703

**Published:** 2026-09-01

**Authors:** Ronald Danny Nyatuka, Pazion Cherinet, Md Shafiqur Rahman Jabin

**Affiliations:** 1 School of Computing and Engineering Sciences (SCES), Strathmore University, Nairobi, Kenya; 2 Orbit Health, Addis Ababa, Ethiopia; 3 Department of Medicine and Optometry, Linnaeus University, Kalmar, Sweden

**Keywords:** digital transformation, evaluation framework, health systems strengthening, implementation science, infrastructure resilience, socio-technical systems, sustainable health systems, user-centered design

## Abstract

Digital health technologies have become integral to health system strengthening, yet their implementation in low-resource settings continues to face persistent challenges related to unreliable infrastructure, limited technical capacity, and constrained financial resources. Although existing digital health evaluation frameworks have advanced understanding of technology adoption, implementation, and sustainability, they frequently treat infrastructure as a contextual factor rather than a core determinant of digital health system performance. This Perspective argues that future evaluation should move beyond technology-centered assessment toward infrastructure-aware evaluation that explicitly recognizes the interdependencies among technical, infrastructural, operational, and economic dimensions. We propose an infrastructure-aware framework for evaluating digital health systems that integrates mixed-methods evaluation and builds upon established implementation science by recognizing infrastructure as a core evaluation domain alongside technical, operational, and economic considerations. The framework positions infrastructure, including energy reliability, internet connectivity, hardware availability, maintenance capacity, and technical support, as a central determinant of implementation feasibility and long-term sustainability. It is informed by implementation science, socio-technical systems thinking, and practical insights from digital health implementation in resource-constrained settings and is illustrated using the Bright Health program in Kenya and Ethiopia. By providing a structured, infrastructure-aware perspective, the proposed framework complements existing implementation science approaches and offers a more comprehensive basis for evaluating digital health systems in low-resource environments. Future research should operationalize the proposed domains, develop standardized evaluation indicators, and empirically validate the framework across diverse technologies and implementation contexts to support more resilient, equitable, and sustainable digital health systems.

## Introduction

Digital health technologies have become central to health system transformation, improving healthcare delivery through electronic health records, clinical decision support systems, telemedicine, mobile health applications, and artificial intelligence. Over the past decade, governments and international organizations have invested substantially in digital health to strengthen health systems, improve continuity of care, and advance universal health coverage (1–4). These investments have been particularly important in low- and middle-income countries (LMICs), where digital technologies help address shortages in the healthcare workforce, infrastructure, and access to specialized services (4–6).

Despite rapid technological advances, evidence on the effectiveness and sustainability of digital health interventions remains inconsistent. Although many initiatives demonstrate promising pilot outcomes, relatively few achieve long-term integration into routine healthcare delivery or sustainable scale-up (7–9). Implementation failures rarely result from technology alone but reflect interactions among technology, healthcare organizations, users, workflows, and the broader infrastructural environment (10–12).

A major yet underexplored challenge is the evaluation of digital health interventions. Existing frameworks assess usability, implementation outcomes, clinical effectiveness, and economic performance, often overlooking the infrastructural conditions that determine whether digital systems operate effectively (13, 14). This limitation is particularly evident in LMICs, where unreliable electricity, intermittent internet connectivity, limited maintenance capacity, and shortages of technical expertise remain persistent barriers (15, 16). Consequently, evaluations focused primarily on technological performance risk overestimating intervention success while underestimating the structural dependencies influencing long-term sustainability.

Recent advances in implementation science and socio-technical systems research recognize digital health interventions as complex adaptive systems rather than standalone technologies (7, 11, 17). Likewise, mixed-methods research integrates quantitative measures of system performance with qualitative evidence on user experience, organizational readiness, and contextual influences (18, 19). However, although some maturity assessment tools, such as the Stages of Continuous Improvement (SOCI) model, include ICT infrastructure as an assessment domain, widely used implementation science frameworks (e.g., CFIR, NASSS, NPT, and RE-AIM) generally position it within broader organizational or contextual constructs rather than as an explicit interacting evaluation domain (6, 13, 20, 21). Building on these approaches, our framework explicitly integrates technical, infrastructural, operational, and economic domains within a mixed-methods evaluation of digital health systems.

This Perspective argues for moving digital health evaluation beyond technology-centered assessment toward infrastructure-aware evaluation. We propose an infrastructure-aware mixed-methods framework for evaluating digital health systems that complements existing implementation science by recognizing infrastructure as a core evaluation domain in low-resource settings. The following sections examine current advances and remaining challenges, present the framework, and discuss its implications for research, implementation, and policy.

## Current advances and remaining challenges in digital health evaluation

Over the past decade, substantial progress has been made in developing frameworks to evaluate digital health interventions. Advances in implementation science, health informatics, and digital medicine have shifted evaluation beyond technical performance toward implementation processes, user adoption, organizational readiness, and health outcomes (7, 11). Frameworks including the Nonadoption, Abandonment, Scale-up, Spread, and Sustainability (NASSS) framework, the Consolidated Framework for Implementation Research (CFIR), and Normalization Process Theory (NPT) have improved understanding of why digital health interventions succeed or fail within complex healthcare systems (10, 11, 13).

These frameworks recognize that successful implementation extends beyond technological functionality. Organizational culture, stakeholder engagement, implementation readiness, workflow integration, and contextual adaptation have become central to digital health evaluation (9, 22, 23). Likewise, mixed-methods research combines quantitative measures of system performance with qualitative insights into user experiences, organizational processes, and contextual influences (18, 19), strengthening methodological rigor and enabling more comprehensive evaluation.

Despite these advances, implementation science frameworks conceptualize infrastructure differently. Frameworks such as CFIR, NASSS, NPT, and RE-AIM primarily address infrastructure within broader organizational, implementation, or contextual constructs rather than evaluating it as an explicit, interacting domain of digital health system evaluation (7, 10, 11). In contrast, maturity assessment tools such as the Stages of Continuous Improvement (SOCI) model include ICT infrastructure as a dedicated assessment domain. However, these tools primarily assess digital health maturity rather than implementing multidimensional evaluation across technical, infrastructural, operational, and economic domains. This distinction is particularly important in low- and middle-income countries, where unreliable electricity, intermittent internet connectivity, constrained financial resources, and limited technical expertise directly affect implementation feasibility and long-term sustainability (15, 16, 20). As a result, theoretical evaluation models often fail to reflect real-world implementation.

Recent implementation experience suggests that infrastructure should be viewed not as background context but as an active component of the digital health ecosystem. Digital technologies interact continuously with organizational processes, users, and physical infrastructure. Consequently, weaknesses in components such as energy reliability or network connectivity can compromise overall system performance, even in well-designed digital solutions (1, 3, 24). This systems perspective supports recognizing infrastructure as a core determinant of implementation success rather than an external contextual variable.

The next generation of digital health evaluation frameworks should move beyond technology-centered assessment toward infrastructure-aware evaluation. Such an approach should integrate technical performance, infrastructural readiness, operational feasibility, and economic sustainability within a unified mixed-methods framework for resource-constrained settings. Rather than replacing existing implementation science frameworks, this perspective complements and extends them by incorporating infrastructure as a core evaluation domain.

**Figure 1:**
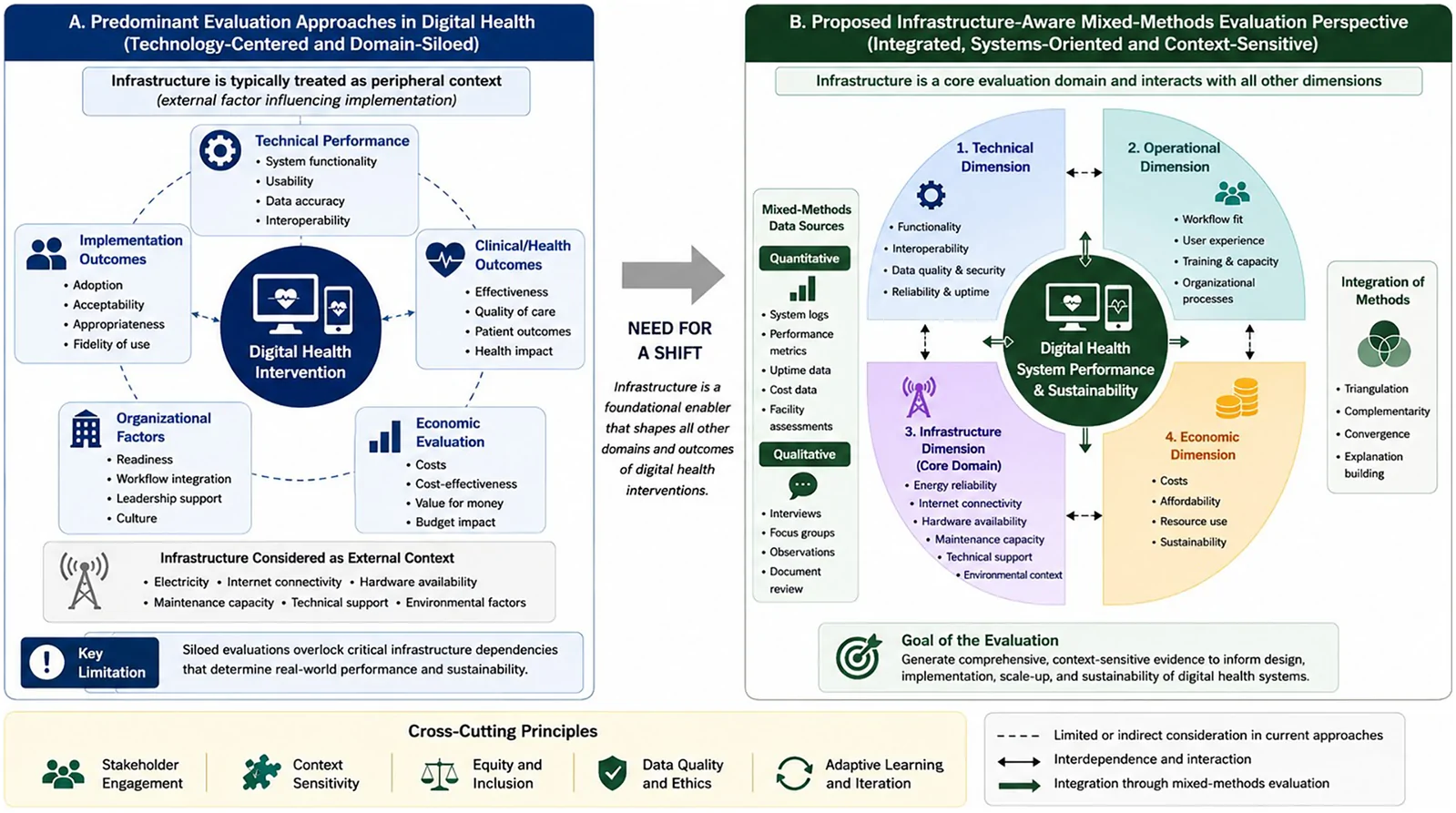
(**A**) Predominant evaluation approaches in digital health (technology-centred and domain-siloed); and (**B**) Proposed infrastructure-aware mixed-methods evaluation perspective (integrated, systems-oriented and context-sensitive).

The conceptual differences between prevailing digital health evaluation approaches and the proposed infrastructure-aware perspective are summarized in Figure 1.

Frameworks such as CFIR, NASSS, NPT, and RE-AIM primarily address infrastructure through broader organizational or contextual constructs, whereas the proposed framework evaluates infrastructure as an explicit domain interacting with technical, operational, and economic dimensions.

## Toward an infrastructure-aware framework for evaluating digital health systems

### Guiding principles for infrastructure-aware digital health evaluation

The next generation of digital health evaluation frameworks should move beyond assessing technology as an isolated intervention to recognizing digital health systems as complex socio-technical ecosystems embedded within organizational and infrastructural environments. Evaluation should assess not only whether an intervention functions as intended but also whether conditions for sustained implementation exist. These include reliable energy supply, digital connectivity, technical maintenance capacity, organizational readiness, governance, and financial sustainability, all of which influence system performance and long-term adoption (7, 10, 11, 25).

A key principle of the framework is that infrastructure should be treated as a measurable component of digital health evaluation rather than a background contextual factor. In low-resource settings, deficiencies in electricity, internet connectivity, hardware availability, and technical support directly affect data quality, workflow continuity, user confidence, and service reliability. Ignoring these dependencies risks overly optimistic evaluations while overlooking practical constraints on implementation success (15, 16, 26).

A second principle is integrating quantitative and qualitative evidence to capture system performance and implementation context. Quantitative indicators, including system uptime, connectivity availability, data completeness, and operational costs, provide objective measures of technical performance but cannot explain why implementation succeeds or fails. Qualitative evidence, including stakeholder experiences, organizational processes, implementation barriers, and contextual adaptations, explains observed performance and supports interpretation of quantitative findings (18, 19, 26).

Finally, digital health evaluation should be adaptive rather than static. As digital health systems evolve, evaluation frameworks should accommodate changing technologies, implementation contexts, and health system priorities. This aligns with implementation science, which recognizes that successful implementation depends on continuous learning, iterative refinement, and responsiveness to local conditions rather than one-time assessment (10, 22, 26). Collectively, these principles underpin the proposed framework.

### Core evaluation domains

The framework comprises four interdependent evaluation domains: technical, infrastructural, operational, and economic. In this framework, the infrastructural domain refers to the enabling physical and organizational resources required to support digital health systems, including reliable electricity, internet connectivity, hardware availability, maintenance capacity, and technical support. In contrast, the technical domain evaluates the functionality and performance of the digital health system itself, including software functionality, interoperability, system reliability, and usability (7, 11, 27).

The technical domain evaluates system functionality and performance, including reliability, interoperability, data quality, cybersecurity, scalability, and resilience. Traditional evaluations emphasize these indicators because they provide measurable evidence of system performance. However, technical performance alone provides only a partial understanding of implementation success, particularly in resource-constrained environments (3, 13).

The infrastructural domain represents the principal innovation of the framework. It includes the physical and digital resources required to sustain digital health implementation, including electricity, renewable energy, internet connectivity, hardware reliability, maintenance capacity, and local technical support. Unlike existing approaches, infrastructure is evaluated as an independent domain because it determines the availability, continuity, and sustainability of digital health services. Infrastructure failures can interrupt healthcare delivery, compromise data integrity, and undermine user trust, particularly in low-resource settings (1, 15, 16, 26).

The operational domain examines how digital health systems integrate into routine healthcare delivery, including workflow compatibility, organizational readiness, workforce capacity, training, governance, user acceptance, and change management. Sustainable implementation depends not only on technical performance but also on integration into routine clinical practice. Implementation science consistently identifies organizational and human factors as key determinants of technology adoption (11, 22, 28).

The economic domain considers financial sustainability across the lifecycle of digital health interventions. Beyond implementation costs, evaluation should include infrastructure investment, maintenance, equipment replacement, workforce development, technical support, and long-term operational expenditure. Many digital health initiatives fail not because technologies lack effectiveness but because financing is insufficient to sustain routine operation and future scale-up (8, 9, 26).

These domains form an interconnected system in which infrastructure enables technical functionality, technical performance influences operational efficiency, operational practice affects economic sustainability, and financial investment supports infrastructure development. Understanding these relationships moves evaluation beyond isolated performance metrics toward implementation readiness and long-term sustainability. Future evaluations should adopt mixed-methods approaches to capture these multidimensional interactions across implementation contexts.

**Figure 2:**
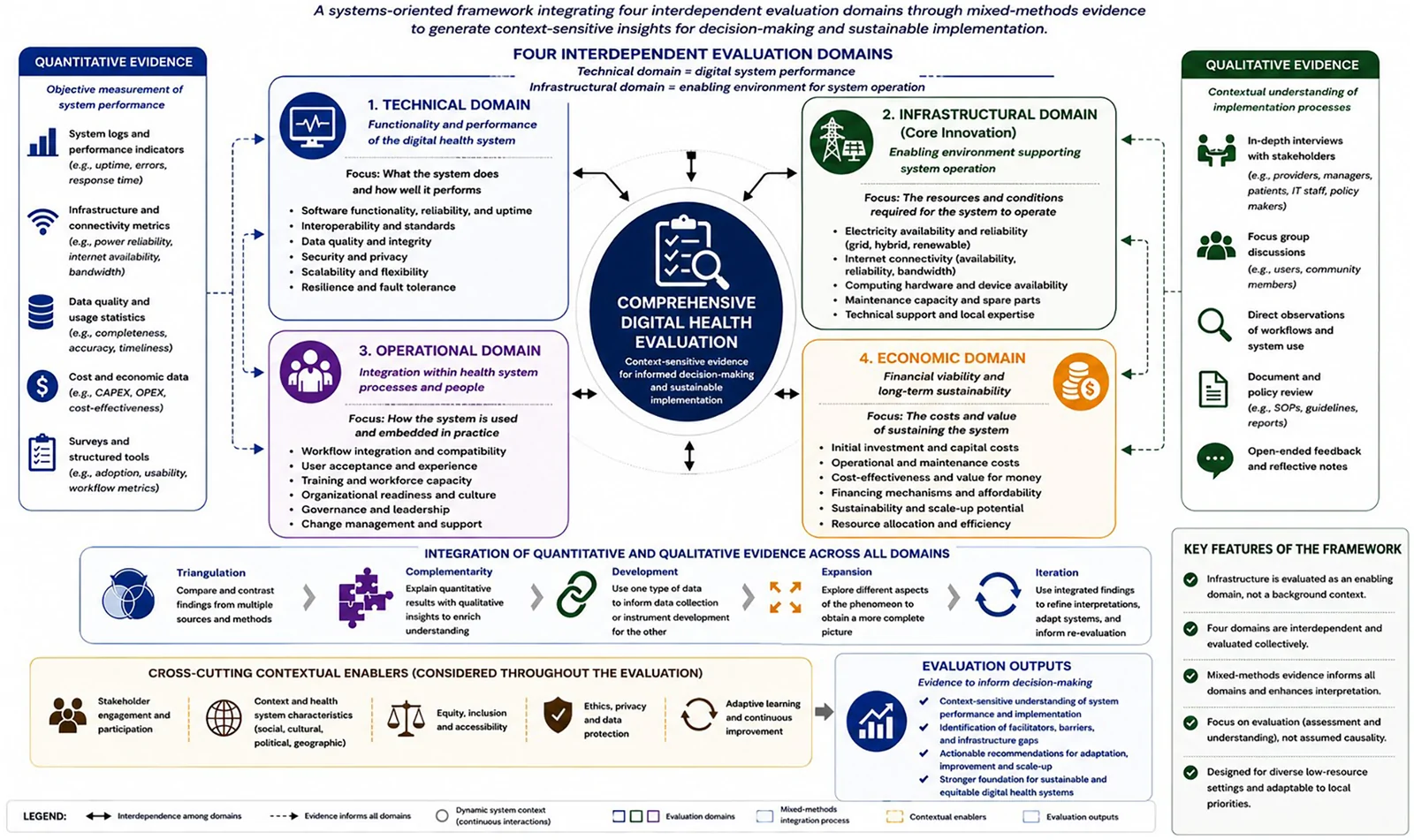
Proposed infrastructure-aware mixed-methods evaluation framework for digital health systems in low-resource settings. presents the proposed infrastructure-aware mixed-methods evaluation framework. The framework distinguishes the technical domain, which evaluates digital system functionality and performance, from the infrastructural domain, which evaluates the enabling environment required for sustained system operation, while integrating operational and economic considerations within a dynamic system context.

Figure 2 illustrates the proposed infrastructure-aware mixed-methods framework, highlighting the four interdependent evaluation domains, their interactions, and the integration of qualitative and quantitative evidence for comprehensive evaluation of digital health systems in low-resource settings.

The framework comprises four interdependent domains, i.e., technical, infrastructural, operational, and economic, integrated through qualitative and quantitative evidence. The technical domain evaluates digital system functionality and performance, whereas the infrastructural domain evaluates the enabling environment required for sustained system operation.

### Integrating quantitative and qualitative evidence

A defining feature of the framework is integrating quantitative and qualitative evidence in digital health evaluation. Digital health systems are complex interventions operating within dynamic healthcare environments where technical performance, organizational processes, infrastructure, and human behavior interact continuously. Consequently, a single methodological approach is unlikely to provide a comprehensive understanding of implementation success or failure. Mixed-methods evaluation addresses this limitation by combining objective performance measures with contextual insights that explain how and why implementation outcomes occur (6, 18, 29).

Within the framework, quantitative evidence provides measurable indicators across the four evaluation domains, including system availability and uptime, network reliability, data completeness and accuracy, workflow efficiency, implementation costs, and resource utilization. These indicators enable performance evaluation and comparisons across facilities, implementation phases, and geographical settings (13, 14, 30). However, quantitative metrics alone cannot explain the organizational, behavioral, and contextual factors shaping observed performance.

Qualitative evidence complements these measures by examining stakeholder experiences, implementation processes, governance, organizational readiness, and contextual influences. Methods such as semi-structured interviews, focus group discussions, observations, and document reviews provide insights into barriers, facilitators, user perceptions, and adaptive strategies often overlooked by quantitative indicators (7, 19). These insights are particularly valuable in low-resource settings, where implementation frequently requires context-specific adaptations not captured by routine performance metrics.

Rather than viewing quantitative and qualitative evidence as parallel streams, the framework emphasizes their integration through established mixed-methods strategies, including triangulation, complementarity, development, expansion, and iterative learning (31–33). Quantitative findings may identify implementation challenges or unexpected performance patterns, while qualitative evidence explains their underlying mechanisms and context. Conversely, qualitative insights can inform the selection of quantitative indicators and identify emerging issues requiring further measurement. This iterative process provides a richer understanding of digital health implementation than either approach alone.

By embedding mixed-methods integration across all evaluation domains, the framework moves beyond determining whether digital health systems function to understanding why they perform as they do, under what conditions they are most effective, and how implementation can be improved. This approach provides a stronger foundation for designing, evaluating, and scaling digital health interventions in resource-constrained settings while supporting evidence-informed decision-making by researchers, implementers, and policymakers.

## Illustrative application in low-resource settings

To illustrate the practical utility of the framework, we present a conceptual application to digital health implementation in low-resource settings. Rather than reporting findings from an empirical mixed-methods study, this illustration demonstrates how the framework can support comprehensive evaluation and evidence-informed decision-making throughout the planning, implementation, monitoring, and scale-up of digital health interventions.

The Bright Health program in Kenya and Ethiopia provides an appropriate context because it integrates digital health technologies with renewable energy solutions to strengthen healthcare delivery in resource-constrained environments (34, 35). Such initiatives operate within complex settings where technological performance depends not only on software functionality but also on infrastructure reliability, organizational readiness, workforce capacity, and financial sustainability. These characteristics make the program well suited to demonstrating an infrastructure-aware evaluation approach.

Within this context, the technical domain could evaluate system functionality, interoperability, data quality, cybersecurity, and service availability. Indicators such as system uptime, synchronization success, data completeness, and application performance provide quantitative evidence of system reliability. The infrastructural domain would assess electricity reliability, renewable energy performance, internet connectivity, hardware availability, maintenance capacity, and local technical support. Evaluating these domains together helps determine whether technical performance reflects software quality, infrastructural constraints, or their interaction.

The operational domain could examine integration into routine healthcare delivery by assessing workflow compatibility, workforce capacity, organizational readiness, governance, and user experiences. Qualitative methods, including interviews, focus group discussions, observations, and document review, provide insights into implementation barriers, adaptive strategies, and contextual influences not captured by routine performance indicators. The economic domain could assess implementation costs, operational expenditure, maintenance requirements, financing mechanisms, affordability, and long-term sustainability to inform resource allocation and investment decisions.

Importantly, the framework integrates evidence across the four domains rather than evaluating each independently. For example, reduced system availability may reflect unstable electricity, limited internet connectivity, insufficient workforce training, or inadequate maintenance rather than a technical problem. Likewise, improvements in technical performance may not translate into sustainable implementation if financing cannot support long-term operation. Considering these interdependencies provides a more comprehensive understanding of implementation challenges and supports context-sensitive solutions.

Although this illustration draws on implementation experience from the Bright Health program, the framework is intended to be adaptable rather than program-specific. Its domains and mixed-methods principles can be applied across digital health initiatives, including electronic health records, telemedicine, mobile health applications, clinical decision support systems, and artificial intelligence–enabled technologies. The framework is intended as a conceptual guide for future evaluation rather than a prescriptive methodology, allowing adaptation to local priorities, available resources, and health system contexts.

## Discussion

### Advancing digital health evaluation through infrastructure-aware thinking

Digital health implementation is increasingly recognized as a complex socio-technical process requiring coordinated interactions among technology, healthcare organizations, users, and broader implementation environments (7, 11). Existing implementation science frameworks, including CFIR, NASSS, and RE-AIM, have advanced understanding of technology adoption, implementation, and sustainability by emphasizing organizational readiness, stakeholder engagement, contextual influences, and implementation processes (6, 10, 36). However, infrastructure has largely remained embedded within broader contextual domains rather than being explicitly conceptualized as a measurable component of digital health evaluation.

This represents an area where existing implementation science and maturity assessment approaches can be further complemented. Digital health systems cannot be fully understood without considering the infrastructural conditions that enable or constrain their operation. In many low-resource settings, reliable electricity, stable internet connectivity, appropriate hardware, and sustainable technical support are prerequisites for routine system functionality rather than peripheral contextual factors. Rather than proposing an alternative implementation theory, the framework extends existing evaluation approaches by recognizing infrastructure as a first-order determinant of implementation feasibility and long-term sustainability.

This perspective also contributes to ongoing discussions on user-centered design (UCD) in digital health. Traditional UCD approaches emphasize usability, workflow integration, and alignment with user needs at the interface and service levels. However, user experience in resource-constrained settings is also shaped by infrastructural conditions that influence system availability, continuity, and reliability. Incorporating energy reliability, connectivity, and maintenance capacity into evaluation broadens UCD toward an infrastructure-aware approach that better reflects digital health implementation in low-resource settings. Future research should operationalize and empirically validate the framework across diverse implementation settings.

### Implications for research, policy, and future framework development

The proposed framework has several implications for digital health research and implementation. First, it encourages comprehensive evaluation strategies that integrate quantitative system indicators with qualitative evidence on organizational processes, user experiences, and contextual influences. Such mixed-methods approaches provide a more complete understanding of implementation than either quantitative or qualitative methods alone (18, 32). Second, the framework offers implementers and policymakers a structured approach to identifying infrastructure-related risks during the planning, deployment, monitoring, and scale-up of digital health programs. This may support more informed investment decisions and strengthen the long-term sustainability of digital health initiatives in resource-constrained settings.

Although informed by implementation experience in Kenya and Ethiopia, the framework is intended to be applicable across diverse low-resource settings. Nevertheless, implementation priorities and infrastructural constraints vary across health systems, and the framework should therefore be adapted to local contexts rather than applied prescriptively. Future research should focus on operationalizing the evaluation domains, developing standardized indicators, and empirically validating the framework across different digital health technologies and implementation environments. Comparative evaluations of electronic health records, telemedicine, mobile health applications, artificial intelligence, and decision-support systems would help refine the framework and establish its practical utility. Continued collaboration among implementation scientists, health informaticians, engineers, policymakers, and frontline healthcare professionals will be essential for advancing infrastructure-aware evaluation and supporting resilient, equitable, and sustainable digital health systems worldwide.

## Conclusions

Digital health evaluation should move beyond technology-centered assessment toward infrastructure-aware evaluation that recognizes the interdependence of technical, infrastructural, operational, and economic domains. This Perspective proposes a mixed-methods framework that complements existing implementation science by explicitly positioning infrastructure as a core component of digital health system evaluation, particularly in low-resource settings. Rather than serving as a prescriptive methodology, the framework is intended to guide comprehensive, context-sensitive evaluation and support evidence-informed implementation, policy, and investment decisions. Future research should operationalize the proposed domains, develop standardized indicators, and empirically evaluate the framework across diverse digital health technologies and implementation settings.

## References

[1] Labrique A Vasudevan L Mehl G Rosskam E Hyder AA. Digital health and health systems of the future. Glob Health Sci Pract. (2018) 6(Suppl 1):S1–4. 10.9745/GHSP-D-18-00342 30305334 PMC6203414

[2] McCool J Dobson R Whittaker R Paton C. Mobile health (mHealth) in low- and middle-income countries. Annu Rev Public Health. (2022) 43:525–39. 10.1146/annurev-publhealth-052620-093850 34648368

[3] Bates DW Levine D Syrowatka A Kuznetsova M Craig KJT Rui A. The potential of artificial intelligence to improve patient safety: a scoping review. NPJ Digit Med. (2021) 4(1):54. 10.1038/s41746-021-00423-6 33742085 PMC7979747

[4] Dehghan M Behzadi A Mehrolhassani MH Ghaemi MM. Challenges and facilitators of electronic health record implementation: a scoping review. Int J Med Inf. (2026) 205:106094. 10.1016/j.ijmedinf.2025.106094 40916274

[5] Mehl G Labrique A. Prioritizing integrated mHealth strategies for universal health coverage. Science. (2014) 345(6202):1284–7. 10.1126/science.1258926 25214614

[6] O'Connor L Dunkel L Weitz AC Walkey A Lindenauer PK Soni A. Digitally Delivered, Systemically Challenged: A Qualitative Study of Health System Readiness for Digital Care. medRxiv (2025). 10.1371/journal.pdig.0001193 PMC12773818 41493964

[7] Greenhalgh T Wherton J Papoutsi C Lynch J Hughes G A'Court C. Beyond adoption: a new framework for theorizing and evaluating nonadoption, abandonment, and challenges to the scale-up, spread, and sustainability of health and care technologies. J Med Internet Res. (2017) 19(11):e8775. 10.2196/jmir.8775 PMC5688245 29092808

[8] Tomlinson M Rotheram-Borus MJ Swartz L Tsai AC. Scaling up mHealth: where is the evidence? PLoS Med. (2013) 10(2):e1001382. 10.1371/journal.pmed.1001382 23424286 PMC3570540

[9] Shaw J Agarwal P Desveaux L Palma DC Stamenova V Jamieson T. Beyond “implementation”: digital health innovation and service design. NPJ Digit Med. (2018) 1(1):48. 10.1038/s41746-018-0059-8 31304327 PMC6550242

[10] Greenhalgh T Abimbola S. The NASSS framework-a synthesis of multiple theories of technology implementation. Stud Health Technol Inform. (2019) 263(263):193–204. 10.3233/SHTI190123 31411163

[11] Damschroder LJ Reardon CM Widerquist MAO Lowery J. The updated consolidated framework for implementation research based on user feedback. Implement Sci. (2022) 17(1):75. 10.1186/s13012-022-01245-0 36309746 PMC9617234

[12] Jabin MSR. Why health information technology safety problems remain invisible. Front Digit Health. (2026) 8:1785141. 10.3389/fdgth.2026.1785141 42221162 PMC13219026

[13] Murray E Hekler EB Andersson G Collins LM Doherty A Hollis C. Evaluating digital health interventions: key questions and approaches. Am J Prev Med. (2016) 51(5):843–51. 10.1016/j.amepre.2016.06.008 27745684 PMC5324832

[14] Kumar S Nilsen WJ Abernethy A Atienza A Patrick K Pavel M. Mobile health technology evaluation: the mHealth evidence workshop. Am J Prev Med. (2013) 45(2):228–36. 10.1016/j.amepre.2013.03.017 23867031 PMC3803146

[15] Maeder A. The spectrum of needed e-health capacity building–towards a conceptual framework for e-health ‘training’. In: Global Telehealth 2014 (2014), 206, 70 25365673

[16] Wootton R Bonnardot L. Telemedicine in low-resource settings. Front Public Health. (2015):3. 10.3389/fpubh.2015.00003 PMC4300819 25654074

[17] Jabin MSR Nilsson E Nilsson A-L Bergman P Jokela P. Digital health testbeds in Sweden: an exploratory study. Digit Health. (2022) 8:20552076221075194. 10.1177/20552076221075194 35186314 PMC8848084

[18] Palinkas LA Aarons GA Horwitz S Chamberlain P Hurlburt M Landsverk J. Mixed method designs in implementation research. Adm Policy Ment Health. (2011) 38(1):44–53. 10.1007/s10488-010-0314-z 20967495 PMC3025112

[19] O'Cathain A. “Mixed methods research”. In: Pope C Mays N. (eds.) (2019). p. 169–80.

[20] Yilma TM Taddese A Mamuye A Endehabtu BF Alemayehu Y Senay A. Maturity assessment of district health information system version 2 implementation in Ethiopia: current status and improvement pathways. JMIR Med Inform. (2024) 12:e50375. 10.2196/50375 39059005 PMC11316158

[21] Greenhalgh T Papoutsi C. Studying complexity in health services research: desperately seeking an overdue paradigm shift. BMC Med. (2018) 16(1):95. 10.1186/s12916-018-1089-4 29921272 PMC6009054

[22] Proctor E Silmere H Raghavan R Hovmand P Aarons G Bunger A. Outcomes for implementation research: conceptual distinctions, measurement challenges, and research agenda. Adm Policy Ment Health. (2011) 38(2):65–76. 10.1007/s10488-010-0319-7 20957426 PMC3068522

[23] Pan D Nilsson E Rahman Jabin MS. A review of incidents related to health information technology in Swedish healthcare to characterise system issues as a basis for improvement in clinical practice. Health Informatics J. (2024) 30(3):14604582241270742. 10.1177/14604582241270742 39116887

[24] Jabin MSR Wepa D Hassoun A. A case report of system configuration issue in medical imaging due to system upgrade- changes in hardware and software. Front Digit Health. (2024) 6:1371761. 10.3389/fdgth.2024.1371761 39347445 PMC11427759

[25] Jabin MSR. From emergence to recoverability: a sociotechnical control trajectory theory of HIT-related risk. Front Digit Health. (2026) 8:1811981. 10.3389/fdgth.2026.1811981 42535094 PMC13422531

[26] Wittich L Rödiger H Rombey T Brecher A-L Kraft L Sgraja S. Navigating the complexities of digital health technology implementation: a scoping review of barriers and facilitators. Implement Sci Commun. (2026) 7(1):69. 10.1186/s43058-026-00892-4 41782054 PMC13064259

[27] Nakkas H Scott P. A conceptual framework for a successful adoption of digital health systems in developing countries. Stud Health Technol Inform. (2025) 328:377–81. 10.3233/SHTI250742 40588949

[28] Jabin MSR. Why digital health fails silently: a sociotechnical theory of health information technology–related risk. Front Digit Health. (2026) 8:1785086. 10.3389/fdgth.2026.1785086 42245003 PMC13231400

[29] Creswell JW Plano Clark VL. Designing and Conducting Mixed Methods Research. 3rd ed. Thousand Oaks, CA: Sage publications (2017).

[30] Jabin MSR. Operational disruption in healthcare associated with software functionality issue due to software security patching: a case report. Front Digit Health. (2024) 6:1367431. 10.3389/fdgth.2024.1367431 38550716 PMC10973543

[31] Greene JC Caracelli VJ Graham WF. Toward a conceptual framework for mixed-method evaluation designs. Educ Eval Policy Anal. (1989) 11(3):255–74. 10.3102/01623737011003255

[32] Fetters MD Curry LA Creswell JW. Achieving integration in mixed methods designs—principles and practices. Health Serv Res. (2013) 48(6pt2):2134–56. 10.1111/1475-6773.12117 24279835 PMC4097839

[33] Jabin MSR. A qualitative, multi-framework methodology for analysing health information technology–related patient safety incidents. Front Digit Health. (2026) 8:1786358. 10.3389/fdgth.2026.1786358 42294047 PMC13260156

[34] Jabin MSR Nyatuka RD. Bridging the data–power paradox: a conceptual–empirical analysis of solar-powered digital health systems in low-resource settings. Front Digit Health. (2026) 8:1848840. 10.3389/fdgth.2026.1848840 42294048 PMC13260055

[35] Jabin MSR Nyatuka RD. Assessing the feasibility of solar-powered digital health infrastructure in rural and peri-urban Kenya: a mixed-methods study. Front Digit Health. (2026) 8:1848844. 10.3389/fdgth.2026.1848844 42577145 PMC13453787

[36] Glasgow RE Vogt TM Boles SM. Evaluating the public health impact of health promotion interventions: the RE-AIM framework. Am J Public Health. (1999) 89(9):1322–7. 10.2105/AJPH.89.9.1322 10474547 PMC1508772

